# Supplementary material for: Self-organized spatial targeting of contractile actomyosin rings for synthetic cell division
Source: Nat Commun. 2024 Nov 29;15:10415. doi: 10.1038/s41467-024-54807-9 (PMC11607352; doi:10.1038/s41467-024-54807-9)
Supplement: Supplementary file 1 — Supplementary Information [file 41467_2024_54807_MOESM1_ESM.pdf]

Supplementary information for

Self-organized spatial targeting of contractile actomyosin rings for synthetic cell division

María Reverte-López[1], Nishu Kanwa[1], Yusuf Qutbuddin[1], Viktoriia Belousova[1], Marion Jasnin[2], Petra Schwille[1*]

[1]*Department of Cellular and Molecular Biophysics, Max Planck Institute of Biochemistry, Martinsried, D-82152, Germany*

[2]*Helmholtz Pioneer Campus, Helmholtz Munich, Neuherberg, D-85764, Germany; Department of Chemistry, Technical University of Munich, Garching,* D-85748, *Germany*

[*]Corresponding author: schwille@biochem.mpg.de

Supplementary Figures

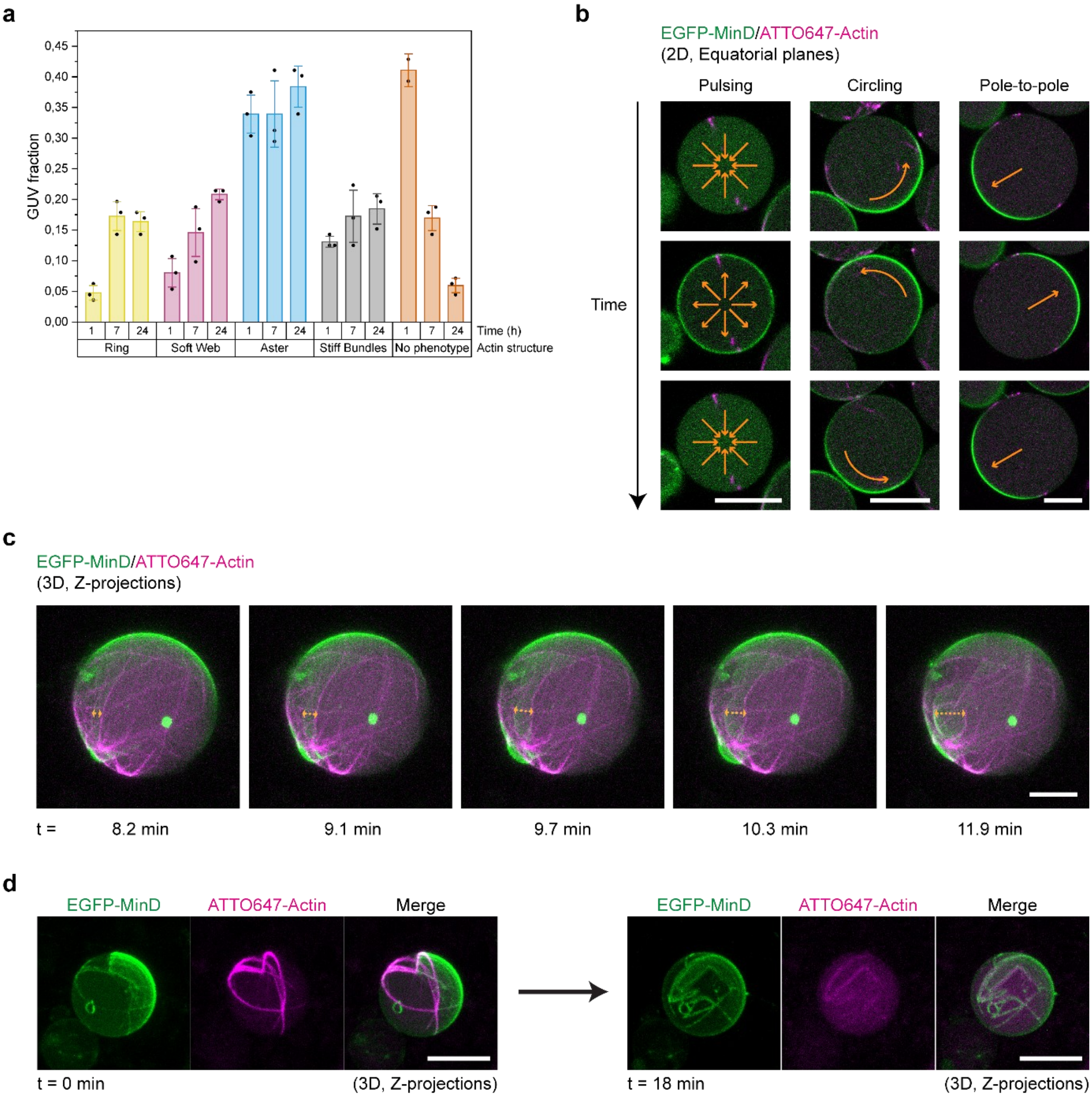

Supplementary Fig. 1 MinDE dynamic oscillations spatiotemporally re-arrange actomyosin bundles via diffusiophoresis. a Bar graph showing the frequencies of actin structures observed inside GUVs at three different timepoints after vesicle production. Inner solution mix: 2.4 µM actin, 0.6 µM fascin (fascin/actin molar ratio = 0.25), 0.05 µM myosin II, 50 g/L Ficoll70, 3 µM MinD, 3 µM MinE and 5 mM ATP. Experiments performed n = 3, total number of GUVs (< 25 µm) analyzed per experiment = 336, number of GUVs analyzed per timepoint = 112. Data shown as mean values with individual data points for each independent experiment. Error bars represent the standard deviation of the 3 experimental runs. **b** 2D confocal images of the three main types of dynamic Min oscillations (pulsing, circling and

pole-to-pole) when the MinDE system is co-reconstituted with actomyosin structures inside vesicles. Orange arrows are meant to represent the direction and dynamic behaviour of the MinDE oscillations. Scale bars: 10 µm. **c** Time-lapse 3D confocal projections of the diffusiophoretic rearrangement of bundles on the vesicle membrane. The chaotic and incremental MinDE binding to areas of the membrane delimited by actomyosin bundles allows the diffusiophoretic transport of neutravidin-bound actin which, in this example, results in the bundles of the soft web being pulled apart (orange dotted arrows). Vesicle inner content: 4 mM actin, 2 mM fascin, 0.05 µM myosin II, 50 g/L Ficoll70, 3 µM MinD, 3 µM MinE and 5 mM ATP. Scale bar: 10 µm. **d** 3D projections of confocal time series showing the collapse of an actomyosin network inside a vesicle induced by the MinDE system. Scale bars: 10 µm. Source data are provided as a Source Data file.

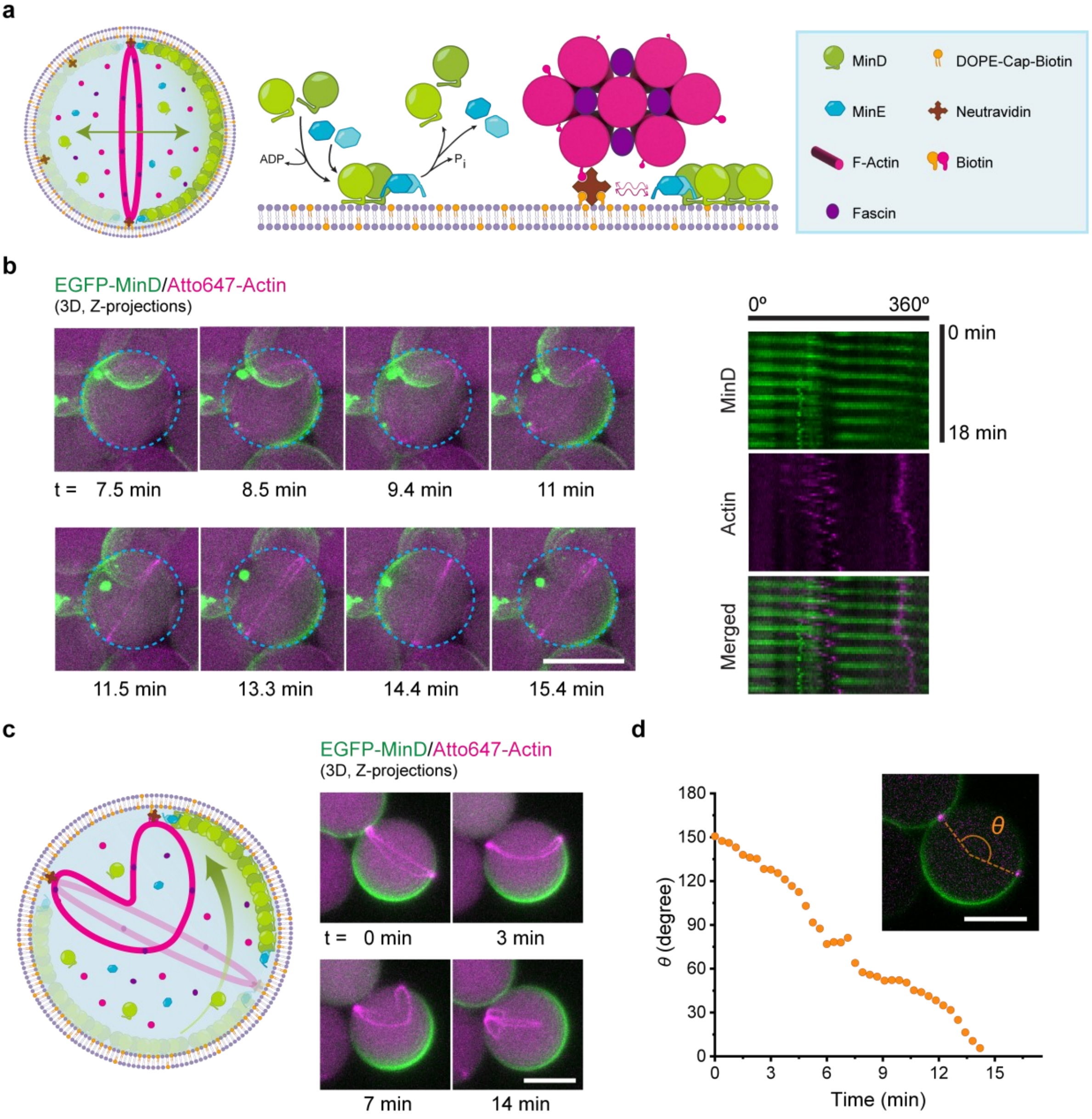

Supplementary Fig. 2 MinDE-driven positioning and buckling of fascin-bundled actin rings inside vesicles. a Schematic illustration of the proteins and molecules employed in encapsulation experiments for sections b and c and the diffusiophoretic effect between actin-fascin bundles and Min proteins at the membrane. **b** 3D projections of confocal time series showing the positioning of a fascin-bundled actin ring by Min proteins. The MinDE pole-to-pole oscillation at the membrane re-orients the ring and locates it perpendicular to the MinDE pattern. Kymographs generated at the GUV equator (blue dotted circle) depict the MinDE-driven change in ring orientation. Vesicle inner content: 1.5 mM actin, 0.3 mM fascin, 10 g/L Ficoll70, 3.2 µM MinD, 1.6 µM MinE and 5 mM ATP. Scale bar: 20 µm. **c** Schematic illustration and 3D projections over time of a vesicle containing an actin ring being folded by the circling oscillation of Min proteins. The MinD protein flux at the rear end of the ring causes

the translocation of one of the ring's endpoints towards its diametrically opposite side. Scale bar: 10 µm. **d** Time course analysis of the angle (θ) between the two endpoints of the ring in section c. After 15 minutes, the counterclockwise MinDE circling pattern co-localizes both endpoints and the ring adopts a folded conformation. Scale bar: 10 µm. Source data are provided as a Source Data file.

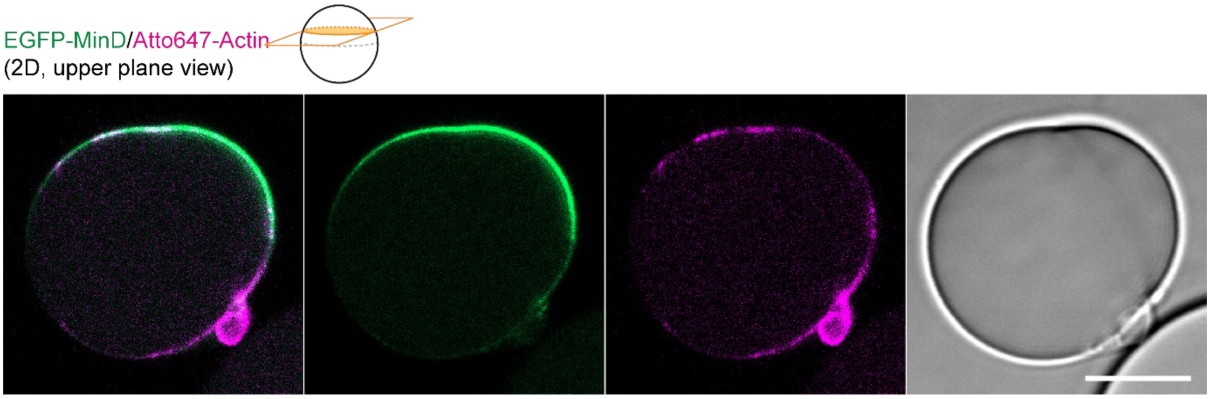

Supplementary Fig. 3 Membrane out-bud formed on a vesicle with a positioned actomyosin soft web. 2D confocal cross-sections from an upper plane of the vesicle in Fig. 3 showing an outward bud attached to the vesicle membrane. Inner solution mix: 2.4 µM actin, 0.6 µM fascin (fascin/actin molar ratio = 0.25), 0.05 µM myosin II, 50 g/L Ficoll70, 3 µM MinD, 3 µM MinE and 5 mM ATP. Scale bar: 10µm.

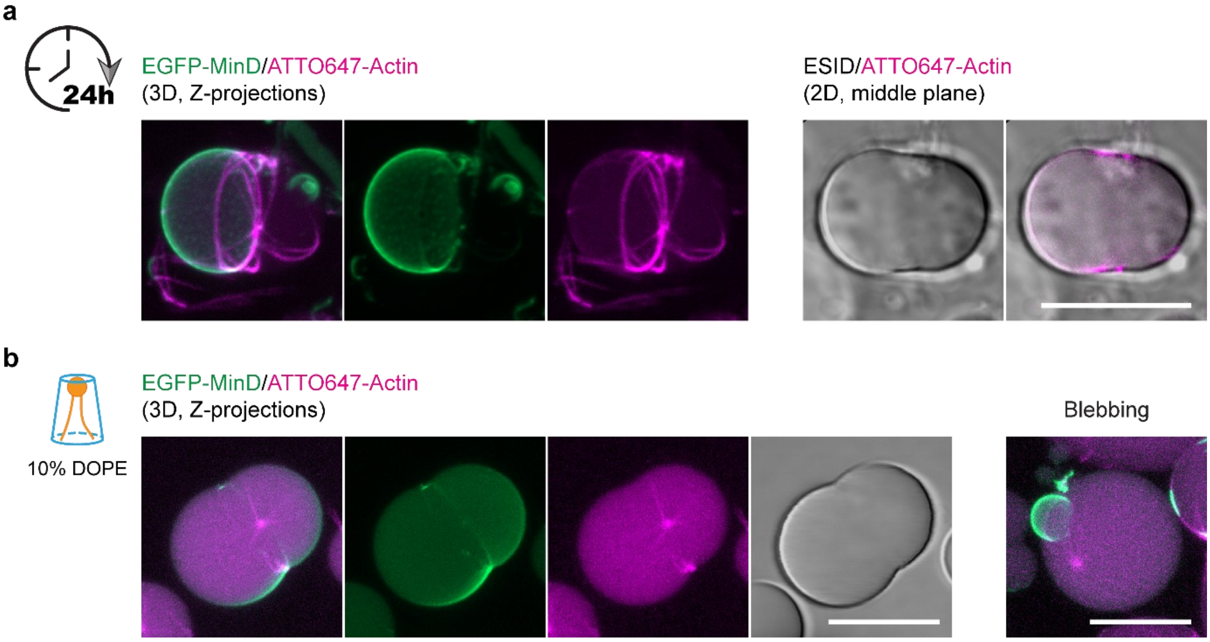

Supplementary Fig. 4 Equatorial deformation and blebbing of vesicles under different experimental conditions. a Confocal images taken 24 hours after encapsulation of a GUV presenting a static MinDE binding to the membrane and a network of actomyosin bundles tightly bound to its furrowed equator (aspect ratio = 0.75). Encapsulating conditions: 2.4 µM actin, 0.6 µM fascin, 0.05 µM myosin II, 50 g/L Ficoll70, 3 µM MinD, 3 µM MinE and 5 mM ATP. Scale bar: 20 µm. **b** Confocal images of two GUVs with a membrane composition consisting of 60% POPC, 30% POPG and 10% DOPE. The deformations and blebbing observed in the presence of this inverse-cone shaped lipid serves as a proof of concept for the addition of other curvature-inducing elements towards contraction enhancement. Inner GUV content as specified in a. Scale bars: 20 µm.

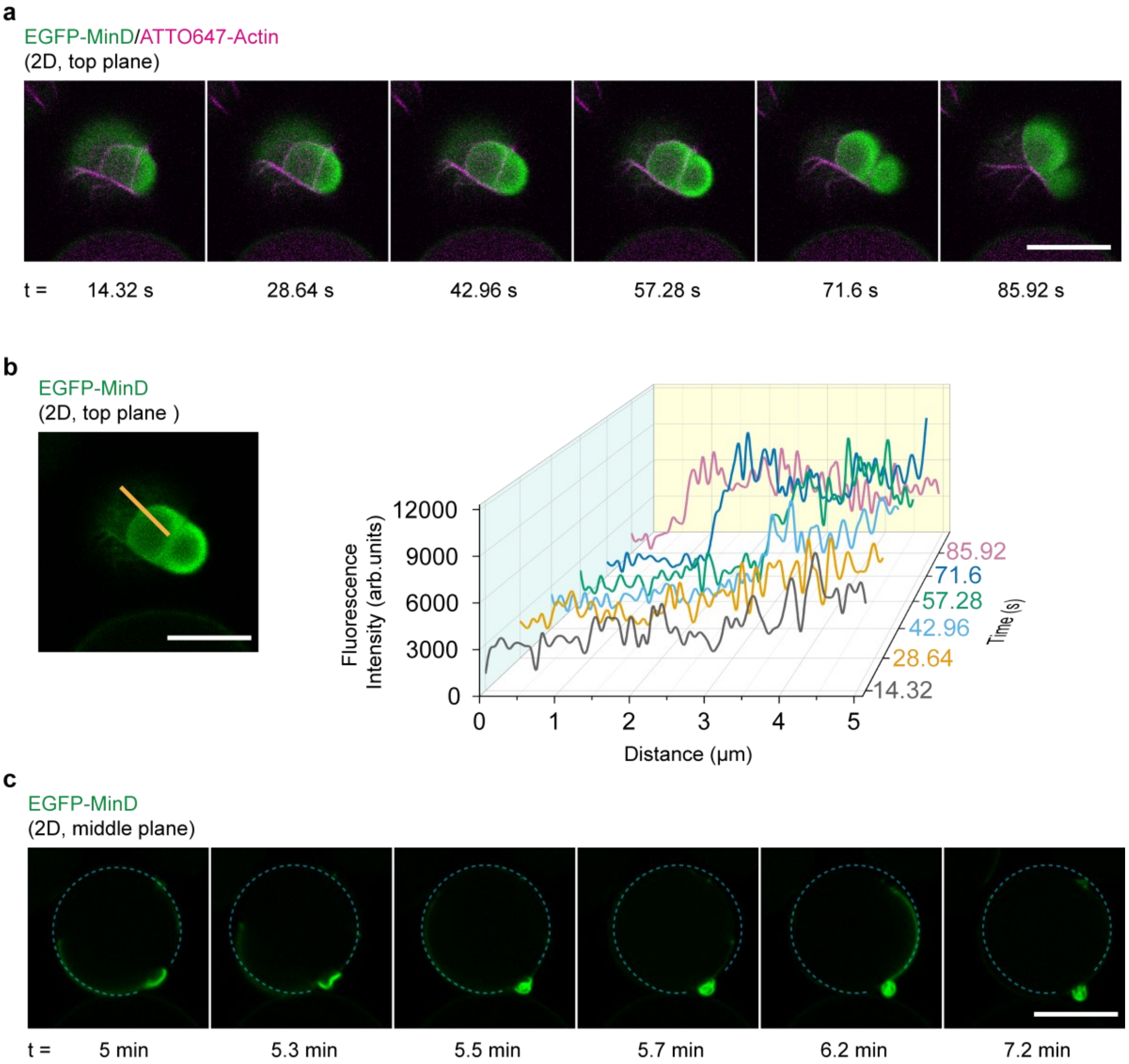

Supplementary Fig. 5 MinDE-induced membrane deformations in vesicles containing reconstituted actomyosin architectures. a Time evolution of bleb initiation on one the forming blebs depicted in Fig. 3. Confocal images of the GUV top plane show how, as Min proteins start binding and accumulating on two regions delimited by actomyosin bundles, outward membrane protrusions form. Encapsulating conditions as specified in Fig. 3. Scale bar: 10 µm. **b** 3D waterfall plot shows the EGFP-MinD fluorescence intensity of a line (orange) drawn over the GUV in section a at six different time points. As the bleb grows with time, the EGFP-MinD fluorescence intensity at the inside of the area delimited by actomyosin bundles increases. Simultaneously, the line section that falls outside of the actomyosin-delimited compartment shows a gradual decline in EGFP-MinD fluorescence intensity over time. Scale bar: 10 µm. **c** Confocal cross-section images at different time points of the vesicle in section a and b which show the formation of a membrane out-bud after blebbing. The outline of the vesicle is depicted as a blue dashed circle. Scale bar: 10 µm. Source data are provided as a Source Data file.

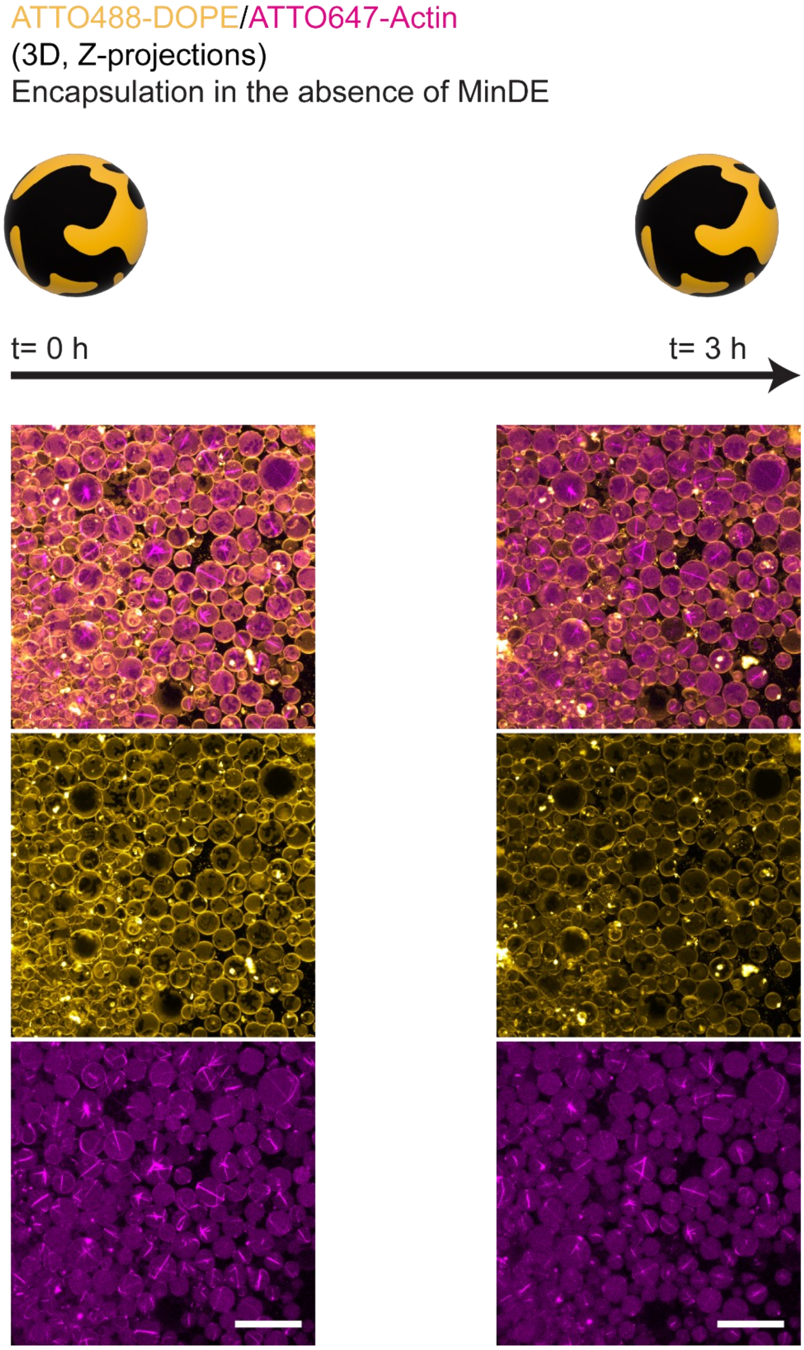

Supplementary Fig. 6 Phase-separated vesicles retain their spherical phenotype in the absence of MinDE proteins. 3D projections of confocal images taken from a three-hour timelapse acquired right after encapsulation. Phase-separated vesicles containing actomyosin networks remain spherical over time and no membrane deformations are observed on the population. Inner encapsulation mix: 2.4 µM actin, 0.6 µM fascin (fascin/actin molar ratio = 0.25), 0.05 µM myosin, 20 g/L Ficoll70 and 5 mM ATP. Encapsulation experiments performed n = 3. Scale bars: 50 µm.
