# Supplementary material for: Self-organized spatial targeting of contractile actomyosin rings for synthetic cell division
Source: Nat Commun. 2024 Nov 29;15:10415. doi: 10.1038/s41467-024-54807-9 (PMC11607352; doi:10.1038/s41467-024-54807-9)
Supplement: Supplementary file 2 — Description of Additional Supplementary Information [file 41467_2024_54807_MOESM2_ESM.pdf]

Description of Supplementary Movies

Note: all movies were generated from Z-stack fluorescence time series by 3D Standard Deviation Z-Projections. The actin channel is depicted in magenta, the MinD channel in green and the brightfield (ESID) channel in greyscale.

File Name: Supplementary Movie 1

Description: MinDE-driven reorganization and positioning of actomyosin bundles at mid-cell. By exploiting the diffusiophoretic transport induced by MinDE proteins, membrane-bound actomyosin bundles are positioned at the equatorial plane of the vesicle, perpendicular to the MinDE pole-to-pole oscillations. Inner content of the vesicle: 2.4 µM actin, 0.6 µM fascin (fascin/actin molar ratio = 0.25), 0.05 µM myosin II, 50 g/L Ficoll70, 3 µM MinD, 3 µM MinE and 5 mM ATP. Timestamp indicates hh:mm:ss. Scale bar: 10 µm.

File Name: Supplementary Movie 2

Description: Constriction of a vesicle at mid-cell by a positioned actomyosin ring. Min pole-to-pole oscillations position an actomyosin ring at the equatorial plane of the vesicle which induces the constriction of the membrane. Stable localization of the ring by Min proteins allows the vesicle to maintain an ellipsoidal shape. Inner content of the vesicle: 4 µM actin, 2 µM fascin (fascin/actin molar ratio = 0.5), 0.05 µM myosin II, 50 g/L Ficoll70, 3 µM MinD, 3 µM MinE and 5 mM ATP. Timestamp indicates hh:mm:ss. Scale bar: 10 µm.

File Name: Supplementary Movie 3

Description: Deformation of a vesicle by a positioned actomyosin soft web. Min pole-to-pole oscillations position the bundles of an actomyosin soft web perpendicular to the Min pattern axis. The strongly anchored actomyosin bundles induce the deformation of the vesicle membrane, breaking GUV spherical symmetry. The membrane out-bud was already present when confocal acquisition started. Inner content of the vesicle: 2.4 µM actin, 0.6 µM fascin, 0.05 µM myosin II, 50 g/L Ficoll70, 3 µM MinD, 3 µM MinE and 5 mM ATP. Timestamp indicates hh:mm:ss. Scale bar: 10 µm.

File Name: Supplementary Movie 4

Description: Deformation of a vesicle by a peripheral actomyosin ring. Vesicle exhibiting Min oscillations which ultimately degenerate to slow circling waves. The constriction of non-positioned actomyosin bundles on one side of the vesicle yields its asymmetric dumbbell shape. Inner content of the vesicle: 4 µM actin, 2 µM fascin, 0.05 µM myosin II, 20 g/L Ficoll70, 3 µM MinD, 3 µM MinE and 5 mM ATP. Timestamp indicates hh:mm:ss. Scale bar: 10 µm.

File Name: Supplementary Movie 5

Description: MinDE-induced blebbing in a vesicle containing a reconstituted actomyosin soft web. MinDE chaotic oscillations on the vesicle membrane induce the deformation of areas delimited by lipid-anchored actomyosin bundles into outward bleb protrusions. The dynamicity of MinDE oscillations is responsible for the growth and retraction of the observed deformations at different areas of the vesicle. Inner content of the vesicle: 2.4 µM actin, 0.6 µM fascin, 0.05 µM myosin II, 50 g/L Ficoll70, 3 µM MinD, 3 µM MinE and 5 mM ATP. Timestamp indicates hh:mm:ss. Scale bar: 10 µm.

File Name: Supplementary Movie 6

Description: MinDE chaotic oscillations on actomyosin networks can generate outward membrane protrusions of different sizes. Example of a vesicle which, over the course of 15 minutes, shows several dynamic membrane deformations of varying scale. Inner content of the vesicle: 4 µM actin, 2 µM fascin, 0.05 µM myosin II, 50 g/L Ficoll70, 3 µM MinD, 3 µM MinE and 5 mM ATP. Timestamp indicates hh:mm:ss. Scale bar: 10 µm.

File Name: Supplementary Movie 7

Description: MinDE-induced bleb deformations on a phase-separated vesicles containing an actomyosin network. Dynamic MinDE oscillations on Ld domains can reorganize membrane-anchored actomyosin bundles, deform Ld domains into outward bleb-like protrusion and remodel the vesicle’s domains. As oscillations progress and actomyosin bundles fold, it is possible to observe the maneuvering and splitting of domains. Inner encapsulation

mix: 2.4 µM actin, 0.6 µM fascin, 0.05 µM myosin II, 20 g/L Ficoll70, 3 µM MinD, 3 µM MinE and 5 mM ATP. Timestamp indicates hh:mm:ss. Scale bar: 10 µm.

File Name: Supplementary Movie 8

Description: Phase-separated vesicles retain their spherical phenotype in the absence of MinDE proteins. Control experiment over 3 hours show that phase-separated vesicles containing actomyosin assemblies, but without Min proteins, maintain their spherical shape and no membrane deformations arise. Inner content of the vesicle**:** 2.4 µM actin, 0.6 µM fascin, 0.05 µM myosin, 20 g/L Ficoll70 and 5 mM ATP Timestamp indicates hh:mm:ss. Scale bar: 20 µm.
